# Novel Synbiotic Yogurt Formulation Supplemented with Fucoidan from *Phaeophyceae* Algae to Promote *Limosilactobacillus reuteri* and *Lacticaseibacillus rhamnosus* GG

**DOI:** 10.3390/foods14152589

**Published:** 2025-07-24

**Authors:** Neus Ricós-Muñoz, Sergi Maicas, Miguel Tortajada-Girbés, Maria Consuelo Pina-Pérez

**Affiliations:** 1 Departamento Microbiologia y Ecologia, Universitat de València, 46100 Burjassot (Valencia), Spain; 2 Pediatric Pulmonology and Allergy Unit, Hospital Universitari i Politècnic La Fe, 46026 Valencia, Spain; 3 Health Research Institute La Fe, 46026 Valencia, Spain; 4 Department of Pediatrics, Obstetrics and Gynecology, School of Medicine, University of Valencia, 46010 Valencia, Spain; 5 Foundation for the Promotion of Health and Biomedical Research in the Valencian Region (FISABIO), 46020 Valencia, Spain

**Keywords:** *Limosilactobacillus reuteri*, *Lacticaseibacillus rhamnosus*, fucoidan, prebiotics, allergy, yogurt, synbiotics

## Abstract

Allergy is recognized as a public health problem with pandemic consequences and is estimated to affect more than 50% of Europeans in 2025. Prebiotic and probiotic food implementation has recently emerged as an alternative strategy to promote immunomodulatory beneficial effects in allergic patients. Among prebiotics, *Phaeophyceae* algae represent a niche of research with enormous possibilities. The present study aims to evaluate the in vitro prebiotic potential of fucoidan from *Fucus vesiculosus*, *Macrocystis pyrifera*, and *Undaria pinnatifida* algae, to promote the growth of *Limosilactobacillus reuteri* and *Lacticaseibacillus rhamnosus* GG as probiotic bacteria added to the formulation of a novel yogurt. Concentrations of fucoidan of 100 and 2000 µg/mL were added to reference growth media and kinetic growth curves for both microorganisms were fitted to the Gompertz equation. Optimized prebiotic conditions for fucoidan were selected to validate in vitro results by means of the formulation of a novel fermented prebiotic yogurt. Conventional yogurts (including *Streptococcus thermophilus* and *Lactobacillus delbrueckii* subs. bulgaricus) were formulated with the different fucoidans, and production batches were prepared for *L. rhamnosus* and *L. reuteri*. Increased *L. reuteri* and *L. rhamnosus* populations in 1.7–2.2 log_10_ cycles just after 48 h of in vitro exposure were detected in fucoidan supplemented yogurt. *M. pyrifera* and *U. pinnatifida* fucoidans were the most effective ones (500 µg/mL) promoting probiotic growth in new formulated yogurts (during the complete shelf life of products, 28 days). Diet supplementation with fucoidan can be proposed as a strategy to modulate beneficial microbiota against allergy.

## 1. Introduction

Nowadays, it is estimated that between 30–40% of the worldwide population is suffering from allergies (among them 150 million EU citizens), and they are expected to affect more than 50% of the European population in 2025 [1,2]. Among the most innovative strategies to combat allergy are recent studies revealing a possible association between the composition, diversity, and stability/dysbiosis of upper-gastrointestinal tract (GUT) microbiome and the development, manifestation, and attenuation of allergy [3,4,5,6,7]. Scientific evidence points out that an altered equilibrium between Firmicutes/Bacteroides can be related to a higher risk of the manifestation of certain types of allergy such as shellfish, nuts, drug, or seasonal pollen allergy [5,8]. In fact, scientific studies carried out in the period 2009–2020, by Canani et al. [9], Chiu et al. [10], Hagan et al. [11], Lee et al. [8], and Yang et al. [12], among others, reveal how a predominance of specific bacterial genera and species in early stages of life (*Clostridium difficile*, *Moraxella catarrhalis*, *Streptococcaceae*, *Porphyromonadaceae*, *Lactobacillaceae*, *Rikenellaceae*, or *Lachnospiraceae*) is related to the subsequent manifestation of specific allergies (atopy development, food allergy, or asthma). According to Hua et al. [5], dysbiosis with a predominance of Bacteroides (e.g., *Bacteroides fragilis*) has been observed to be significatively related to the manifestation of a peanut and tree nut allergy. Chiu et al. [10] also observed a significantly lower abundance of organisms of the phylum Firmicutes in children with asthma and allergic rhinitis than in healthy individuals. An evaluation of fecal microbiota through the different stages of life is leading to an extremely valuable conclusion: although the origin of microbiota dysbiosis is not well understood to date, the conformation of microbiome diversity and stability is specifically crucial and conditioned in infancy from 0 to 3 years old [13,14]. Rodriguez and co-workers named the period comprising pregnancy, birth, and infancy the “window of opportunity for microbiota modulation”. In fact, recent studies are proposing the strategy to target the gut microbiota with probiotics, prebiotics, and dietary modulation as a rational therapeutic approach to prevent allergic diseases [15].

In an infant dietary pattern, yogurt is considered as one of the food products to be introduced first (6–12 months) due to its enormous beneficial properties for children in the first stage of development. It is a vehicle of probiotics, a source of easily digestible milk protein, organic acids, and specific aminoacids, and has the ability to eliminate lactose intolerance symptoms and promote valuable fatty acid richness, among other things [16]. Traditionally, the viable bacteria in yogurt should be integrated with Streptococcus thermophilus and *Lactobacillus delbrueckii* spp. *bulgaricus* (>10^7^ CFU/g). However, the supplementation of yogurt with novel probiotic strains and its viability analysis are being carried out to improve the technological and nutritional properties of these fermented products. Anti-allergic effects have been reported associated with some lactic acid bacteria (LAB), such as in the case of *Lactobacillus rhamnosus* GG, *Bifidobacterium lactis* Bb-12, *Lactobacillus casei*, and *Lactobacillus reuteri* DSM 122460 [17], which, in vitro and in vivo, demonstrated allergy symptomatology attenuation (a downregulation of Th2) [17,18]. Li et al. [15] evaluated the in vivo effect of *Lactobacillus* spp. oral administration against an asthmatic simulated murine model. *Lactobacillus reuteri* resulted as the most effective at alleviating inflammatory symptoms and even decreasing the total IgE of the studied cohort. Also, *Lactobacillus rhamnosus* GG has been defined as a supplementary strategy with promising results supporting sublingual immunotherapy in allergenic children [19].

In parallel, prebiotics such as inulin, fructo-oligosaccharides, or soy oligosides are reported to stimulate the implantation of some beneficial and bifidogenic bacteria, significantly increasing the abundances of Bifidobacteria and Lactobacilli in the colonic tract [20]. The most innovative prebiotics are extracted from algae, and emergently introduced in nutraceutical products, due to their demonstrated functional and recognized health-promoting properties [21,22]. Recent yogurt developments include *Isochrysis galbana* and *Arthrospira platensis* (spirulina) as prebiotics in their formation, reinforcing the functional contribution of yogurt to the human diet [23,24]. Nowadays, the food industry is integrating fucoidan into the formulation of pasta, bread, and beverages, among others, improving organoleptic and nutritional food properties, and promoting the anti-inflammatory, antioxidant, and antimicrobial functional effects of novel food commodities [25]. The contribution of fucoidan in supplemented food products is proposed as a novel strategy to modulate gastrointestinal microbiota [26,27]. Recent studies have remarked on the potential of fucoidan to modulate microbiome and metabolome in human fecal samples [27]. According to the studies of Huan et al. [27], the abundance of *Escherichia-Shigella* and *Klebsiella* decreased in fecal cultures, while the abundance of Bacteroides (*Bifidobacterium*, *Lactobacillus*, and *Megamonas*) increased.

The present study aims to assess the fucoidan (sulphate-fucose polysaccharide from brown algae–*Phaeophyceae*) effect as a prebiotic agent in promoting the in vitro growth of *Lactobacillus reuteri* and *Lactobacillus rhamnosus* GG, the two most promising bacterial species with the potential capability to improve allergenic symptomatology [12,15,17,28,29]. Fucoidan from different algae origins, *Undaria pinnatifida*, *Macrocystis pyrifera*, and *Fucus vesiculosus* will be assessed, and the most promising results obtained in terms of prebiotic potential will be validated on yogurt formulation.

## 2. Materials and Methods

### 2.1. Lactobacillus spp. Strains and Culture

Lyophilized bacterial strains used in the present study were provided by the Spanish Culture Type Collection (CECT) (Paterna, Spain): *L. reuteri* (CECT 925) and *L. rhamnosus* GG (CECT 288). The reactivation and bacterial culture was carried out according to CECT specifications, using sterile Man Rogose Sharp Broth (MRSB, Conda-Pronadisa, Madrid, Spain). Briefly, lyophilized powder was added to 20 mL of sterile MRSB (flasks of 25 mL), and was incubated under anaerobic conditions at 37 °C, for 3 h (anaerobic jars). After that, the inoculum was transferred to 480 mL of sterile MRSB (in 1 L flasks), and incubated afterwards for 48 h under the same optimal conditions. The grown culture was recovered by centrifugation (3000× *g*, 10 min, 25 °C). The supernatant was removed, and the pellet was resuspended in sterile MRSB to carry out a second centrifugation cycle. The final pellet was resuspended in a mixture of sterile suspension MRSB and glycerol [80:20] (*v*/*v*). The final bacterial suspension for each microorganism (*L. reuteri* 3 × 10^9^ CFU/mL; *L. rhamnosus* GG 5 × 10^10^ CFU/mL) was stocked in cryovials (2 mL) at −80 °C.

### 2.2. Fucoidan

Fucoidan is a polysaccharide present in cell walls of *Phaeophyceae* brown algae and also in some marine invertebrates. This chemical compound is characterized as being rich in sulphate groups and L-fucose. Fucoidan from different species was used in the present study: *Fucus vesiculosus* (F8190 Sigma, molecular weight 82 kDa, sulfate content 24.5% (*w*/*w*), Madrid, Spain); *Macrocystis pyrifera* (F8065 Sigma, molecular weight 176 kDa, sulfate content 27% (*w*/*w*)); and *Undaria pinnatifida* (F8315 Sigma, molecular weight 51 kDa, sulfate content 30% (*w*/*w*)) (Merck Life Science S.L.U., Madrid, Spain) [24].

A highly concentrated suspension of fucoidan was prepared (10.000 µg/mL) in sterile miliQ water. This suspension was aliquoted and stored at −80 °C up to the moment of use.

### 2.3. In Vitro Growth Trials Under Fucoidan Exposure

To carry out the *L. reuteri* and *L. rhamnosus* GG in vitro growth experiments, liquid sterile MRSB was used as culture media. Bacterial stocked cultures (at −80 °C) were re-activated in 20 mL of sterile MRSB (dilution 1/1000). Reactivation cultures were incubated 72 h at 28 °C, under anaerobic conditions (anaerobic jars, Thermo Scientific, Waltham, MA, USA). Reactivated aliquots, freshly prepared, were used as initial inoculums to start the in vitro growth trials.

Before bacterial inoculation, different sterile flasks were prepared with both sterile MRSB and sterile MRSB supplemented with fucoidan in order to achieve suspensions with final fucoidan concentrations of 100 and 2000 µg/mL. For the in vitro assays, sterile flasks including 100 mL of sterile MRSB (supplemented/not supplemented with fucoidan) were used as culture media to follow *L. reuteri* and *L. rhamnosus* growth. Bacterial reactivated inoculums were independently added to sterile MRSB media (initial concentration in trials 6.48 ± 0.33 log_10_ cycles). Flasks including MRSB without fucoidan were considered as controls for growth. All inoculated flasks were prepared in triplicate and were incubated at 28 °C for 48 h under anaerobic conditions.

Liquid aliquots (1 mL) were extracted from each inoculated media at time intervals in the range 0–48 h (0, 4, 8, 24, 26, 32, and 48 h). Cell densities were determined based on optical spectrophotometric measurements (OD600 nm) (T60U Spectrometer, PG Instruments Ltd., Leicester, UK) and also by a plate count procedure. In this way, aliquots were serially diluted in order to quantify the bacterial load by viable plate count in MRS agar (MRSA). Bacterial counts were expressed as colony forming units per milliliter (CFU/mL).

### 2.4. Estimation of the Prebiotic Effect of Fucoidan

The prebiotic effect of fucoidan on both *L. reuteri* and *L. rhamnosus* growth was estimated by means of observing the difference between the growth on the reference media (MRSB) and the media supplemented with fucoidan at different studied concentrations (C1 = 100 µg/mL; C2 = 2000 µg/mL), based on the studies of Rubel et al. [30]. The prebiotic potential was estimated at different time intervals (average of three replicates).

(1) $$\mathrm{Prebiotic effect}\;({\mathrm{Log}}_{10}\;\mathrm{cycles growth increment})={Log}_{10\;}\;\left(Nt\;MRSBfucoidan\right)-{Log}_{10\;}(Nt\;MRSB)$$

where *Nt* is the final bacterial load at each period (CFU/mL); *MRSBfucoidan* is the supplemented reference media with fucoidan (from *M. pyrifera*, *U. pinnatifida*, and *F. versiculosus*; concentrations 100–2000 µg/mL)

### 2.5. Mathematical Modelling

Growth kinetic curves (*log*_10_ (CFU/mL) versus time (h)) were obtained and fitted to the Gompertz equation (Equation (1)) according to Belda-Galbis et al. [31]:

(2) $${log\;}_{10}Nt\;=\;A+C\;\times \;e^{-e^{\left(-B\;\times \;\left(t-M\right)\right)}}$$

where the independent variable [*log*_10_ *Nt*] corresponds to the decimal log of bacterial count (CFU/mL) at each time *t*; *A* represents the lower asymptote in the curve (*log*_10_ (CFU/mL); *C* the difference between the curve asymptotes (*log*_10_ CFU/mL); *B* the relative growth rate when *t* = *M* ((*log*_10_ CFU/mL)/h); and *M* the elapsed time until the maximum growth rate is reached (h).

*A*, *B*, *C*, and *M* Gompertz parameters were then used to calculate the lag phase duration (ʎ, h) and the maximum specific growth rate (µmax; (*log*_10_ CFU/mL)/h) reached by *L. reuteri* and *L. rhamnosus* GG under each one of the studied conditions [31].

### 2.6. Fucoidan Yogurt Formulation and Microbiological Analysis

**Figure 1:**
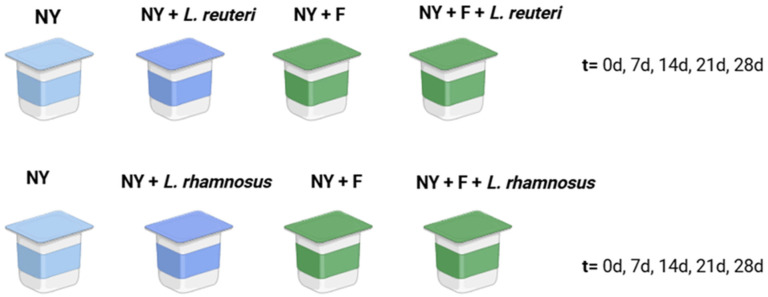
Validation assay of fucoidan prebiotic potential in the development of a probiotic yogurt formulation including *Lacticaseibacillus rhamnosus* GG and *Limosilactobacillus reuteri*, being: NY, natural yogurt, not supplemented with fucoidan, and not including probiotics; NY + F, natural yogurt supplemented with fucoidan; NY + *L. reuteri*, probiotic yogurt including *L. reuteri*; NY + F + *L. reuteri*, probiotic yogurt including *L. reuteri*, supplemented with fucoidan; NY + *L. rhamnosus*, probiotic yogurt including *L. rhamnosus*; NY + F + *L. rhamnosus*, probiotic yogurt including *L. rhamnosus*, supplemented with fucoidan. Yogurts were analyzed just after processing (t = 0 d) and during a refrigeration period of 28 d (2 replicates per batch, 3 batches per sample).

To validate the prebiotic effect associated with different fucoidans (from *U. pinnatifida*, *M. pyrifera*, and *F. vesiculosus*) in the reference media (MRSB) on *L. reuteri* and *L. rhamnosus* growth, different yogurt formulations were prepared at the laboratory (Figure 1) using cow sterilized milk (commercial UHT cow’s milk, fat content 0.5% (*w*/*v*); Pascual S.A. (Madrid, Spain)). Firstly, conventional yogurts were prepared using a lyophilized mixture of *Lactobacillus delbrueckii* subsp. *bulgaricus* and *Streptococcus thermothilus* strains as starters (provided by YOMIX TM (Danisco, Niebüll, Germany)) (2 g starter added per liter of milk) (first batch of yogurts as control). Second and third batches of yogurt were prepared including probiotic strains such as yogurt inoculated with *L. reuteri* (10^2^ CFU/mL initial load) and yogurt inoculated with *L. rhamnosus* (10^2^ CFU/mL initial load). Fourth, fifth, and sixth batches were prepared including control yogurts, *L. reuteri* yogurts, and *L. rhamnosus* GG yogurts, supplemented with the fucoidan at 500 µg/mL concentration Three replicates per batch were prepared on independent days. Two replicated yogurts were prepared for each condition and batch.

The milk mixtures were transferred to 100 mL glass jars, and incubated at 42 °C for 4 h to achieve a pH of 4.3 ± 0.02. pH registration values and representative aliquots for microbiological analysis were periodically taken at 30 min intervals (during the fermentation period). After fermentation, yogurt samples were cooled and transferred to a refrigerator at 4 °C. These samples were analyzed just after bacterial inoculation in milk (0 h), directly after production (4 h, day 0), and after 7, 14, 21 and 28 days of refrigerated storage.

LS Differential Agar (Merck Life Science S.L.U., Madrid, Spain) and M-TRLV agar were used to differentiate Lactobacilli and Streptococci on the basis of colonial morphology, T.T.C. reduction, and casein reaction all over the storage time. Additionally, Man Rogose Shape Agar (MRSA, Scharlab, Barcelona, Spain) was used to validate total bacterial count evolution on the different yogurt formulations.

### 2.7. Statistical Analysis

The software Statgraphics Centurion XVIII (Statgraphics Technologies Inc., The Plains, VA, USA) was used to carry out the statistical analysis of experimental trials. Mean and standard deviation values were obtained for each experimental growth result. An ANOVA analysis was performed to determine the most significant parameters affecting bacterial growth (*p*-value < 0.05). The goodness of Gompertz fit to experimental data was estimated by means of the calculation of Accuracy factor [31].

## 3. Results and Discussion

### 3.1. The In Vitro Fucoidan Prebiotic Effect on L. reuteri and L. rhamnosus Growth

**Figure 2:**
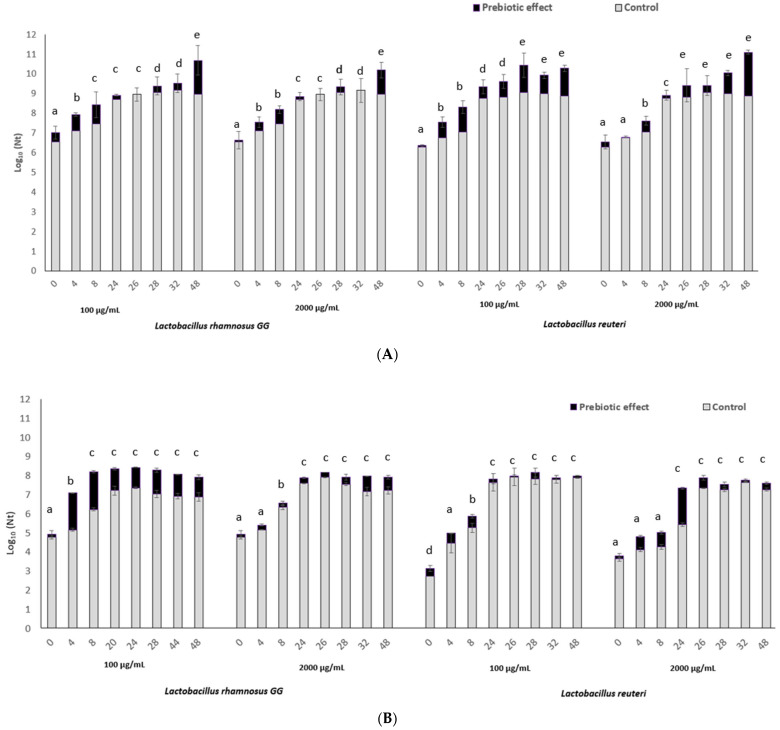

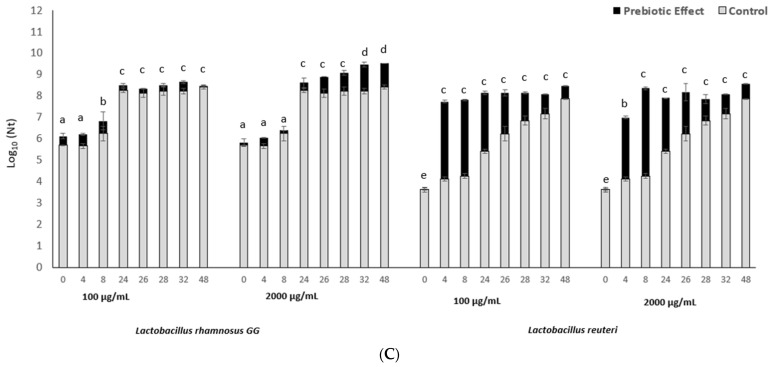
Kinetic growth behavior of *Limosilactobacillus reuteri* and *Lacticaseibacillus rhamnosus* GG in Man Rogose Shape broth (MRSB), supplemented and not supplemented with fucoidan: (**A**) *Fucus vesiculosus*, (**B**) *Undaria pinnatifida,* (**C**) *Macrocystis pyrifera* (being N*t*: final count of bacteria (CFU/mL) registered in growth media at each time period 0–48 h). Superscript letters (a–e) in bars refer to significant differences (*p*-value < 0.05) detected in final bacterial load (Log_10_ Nt) depending on the studied conditions applied (exposure time, fucoidan concentration, and bacteria for each studied fucoidan, (**A**–**C**)).

Figure 2 shows the kinetic growth behavior (log_10_ Nt versus t) (in bars) for both, *L. reuteri* and *L. rhamnosus* GG in MRSB control and supplemented with fucoidan.

As can be seen graphically, the addition of fucoidan, significantly increases the in vitro growth capability of both microorganisms in MRSB (*p*-value < 0.01). The prebiotic potential was dependent on fucoidan origin and fucoidan concentration (*p*-value < 0.05), observing a specific effect for each fucoidan on the in vitro growth behavior of each bacteria.

The fucoidan from *Fucus vesiculosus* algae showed a prebiotic potential equivalent to 2.2. log_10_ cycles growth on *L. reuteri* (C = 2000 µg/mL; exposure time 48 h) (see Figure 2A). Meanwhile, the *Fucus vesiculosus* fucoidan at 2000 µg/mL concentration showed a slightly lower prebiotic potential, equivalent to 1.72 log_10_ cycles on *L. rhamnosus* in vitro growth.

Regarding fucoidan from *Undaria pinnatifida* (Figure 2B), a concentration of 100 µg/mL was enough to reach a prebiotic effect equivalent to 2 log_10_ cycles growth improvement in *L. rhamnosus* GG (just after 4 h of exposure), in comparison with the growth of this bacteria on the MRSB media not supplemented with fucoidan. No significant prebiotic effect was observed on *L. reuteri* growth under the exposure to fucoidan from *Undaria pinnatifida*.

The most significant prebiotic potential was obtained for fucoidan from *M. pyrifera* (Figure 2C), increasing *L. reuteri* bacterial counts in levels closest to 3.5–4.1 log_10_ cycles, after 4–26 h of exposure time.

Increments in bacterial load were between 1 to 4 log_10_ cycles for *L. reuteri* in MRSB supplemented with fucoidan in the range 100–2000 µg/mL. The effect of fucoidan on *L. rhamnosus* GG growth was also significant (*p*-value < 0.01), with 0.95 ± 0.40 log_10_ cycles load increments under 100 µg/mL fucoidan exposure, and 1.75 ± 0.32 log_10_ cycles increment when MRSB was supplemented with fucoidan at 2000 µg/mL.

The fucoidan prebiotic effect increasing *L. reuteri* and *L. rhamnosus* GG growth was significantly affected by both the fucoidan (100–2000 µg/mL) concentration and the exposure time (*p*-value < 0.01). Although the maximum in vitro growth-stimulating effect was observed when fucoidan was added at 2000 µg/mL, for both microorganisms, close to 1 log_10_ cycle of bacterial load increment was observed when fucoidan was added at 100 µg/mL, under an exposure time of 48 h. For *L. reuteri*, a minimum exposure time of 4 h is required to show a significant prebiotic effect attributed to fucoidan supplementation (*M. pyrifera* supplementation). In the case of *L. rhamnosus* GG, the prebiotic effect was also detected just after 4–8 h exposure to fucoidan (*Undaria pinnatifida* supplementation).

Kinetic parameters defining the growth behavior of both bacteria in the MRSB control assays and under the fucoidan exposure were estimated from the Gompertz fitting model. Table 1 includes the values of lag phase duration (ʎ, h) and µmax (log_10_ (CFU/mL)/h) for each studied scenario. Increased maximum specific growth rate (µmax, log_10_ (CFU/mL)/h) values were obtained by means of the addition of fucoidan to the media at different concentrations, there being no significant differences between *L. reuteri* and *L. rhamnosus* GG µmax values at each media, MRSB control, and MRSB fucoidan (100–2000 µg/mL) supplemented media. Regarding lag phase duration, although *L. reuteri* showed a longer ʎ just in the control MRSB media, fucoidan supplementation seems to significantly reduce the lag phase values for both *L. reuteri* and *L. rhamnosus* GG. There is a lack of information defining the relationship between fucoidan origin, structure, and concentration, and its kinetics on antimicrobial, prebiotic, and apoptotic immunomodulatory, which showed effects in vitro and in vivo [28,31]. This aspect is crucial in order to better understand fucoidan functional capability and to define future strategies for use. To our knowledge, the present research work is the first to assess the kinetics of fucoidan in stimulating a growth effect on probiotics.

The in vitro prebiotic potential of vegetables (including soluble dietary fiber, inulin-derived fructans (fructo-oligosaccharides; FOS) and galacto-oligosaccharides (GOS)), and algae materials has been previously described by different authors, pointing out the capability of complex carbohydrates to act as stimulating beneficial microbiota [32,33,34,35]. Pérez-López et al. [35] registered the growth promotion effect of okara, increasing bifidobacterial and lactobacilli in just 48 h of exposure. The effect of an inulin-rich carbohydrate’s prebiotic potential on in vitro *Lactobacillus paracasei* growth was previously studied by Iraporda et al. [33]. Although inulin, a naturally occurring fructooligosaccharide, is widely utilized as a prebiotic for Lactobacilli and Bifidobacteria, Obasola et al. [36] demonstrated no significant effect of this prebiotic on promoting an in vitro growth capability of *L. reuteri* in MRSB supplemented with 5% (*w*/*v*) inulin.

Some prebiotics have been associated not only with stimulating beneficial bacterial growth, but also with the capability to reduce the colonization of harmful bacteria in the gastrointestinal upper tract. In this regard, fucoidan has been described as able to reduce the growth capability of *Helicobacter pylori*, in vitro [37,38] and in vivo [39]. The in vitro and in vivo studied concentrations of fucoidan used as a prebiotic or antimicrobial agent (effective against bacteria and viruses) are limited, and restricted to the interval of minimum 0.1 µg/mL (against canine distemper virus, CDV) to maximum 0.7 g/mL (required dosage against human immunodeficiency viruses, HIV). A 100 µg/mL fucoidan concentration showed a potent antimicrobial in vivo effect against *Helicobacter pylori* using the *Caenorhabditis elegans* model. This previous conclusion could be compared with the results obtained in our study, possibly indicating that 100 µg/mL is a suitable concentration to exert, simultaneously, a probiotic promoting effect (on *L. reuteri* and *L. rhamnosus*) and an inhibitory effect on harmful bacteria (*H. pylori*). According to present results, fucoidan required an in vitro exposure time fixed in 32–48 h to show a significant prebiotic effect. Meanwhile, an in vitro exposure time equal to 24 h was enough to reduce initial *H. pylori* counts in 2 log_10_ cycles [39]. According to Palacios-Gorba et al. [39] and results included in the present study, an in vitro bacterial exposure time of 24–48 h would be effective in increasing beneficial microbiota (≈2 log_10_ cycles for *L. reuteri* and *L. rhamnosus* GG) and reducing pathogenic gastrointestinal upper tract microbiota (≈2 log_10_ cycles).

To date, fucoidan is available only in cosmetics, functional foods, and dietary supplements as a regenerative, anti-inflammatory, and anti-cancer factor for patients with immune-compromised, musculoskeletal, cardiovascular, or gut diseases [40,41]. However, to date there are no registered developed drug products with fucoidan. Studies that assessed the immunomodulatory capability of fucoidan in relation to allergy regulation—either as a direct allergy-modulatory agent (natural ligand for class A scavenger receptors (SR-A), which play critical roles in regulating the body’s innate immune response and adaptive immune responses), or as indirectly stimulating specific probiotics with attenuation effects on allergy manifestations (the severity of an asthmatic crisis, the direction of inflammatory-associated symptoms, an attenuation of atopy and eczema manifestation, and even the reduction of rhinitis) [42,43]—significantly increased during the period 2010–2020. An ovalbumin- (OVA) induced anaphylaxis model (mice) was developed by Mizuno and co-workers [43] to study the allergy-direct-modulatory effect of fucoidan orally administered. According to the results obtained by Mizuno et al. [44], fucoidan was shown to be effective in the suppression of allergic symptoms in sensitized mice by inducing galectin-9 production from colonic epithelial cells. Tian et al. [45] demonstrated the effect of fucoidan in reducing atopy symptomatology. Through fucoidan administration, the infiltration of CD4+ T cells in skin lesions and spleens of atopic dermatitis-affected mice, was significantly reduced compared to control (not treated) mice. The stimulating effect of fucoidan (100–2000 µg/mL) on *L. reuteri* and *L. rhamnosus* GG growth can have a positive impact on patients with milk hypersensitivity (by means of the increased expression of CR1, CR3, FcgRIII, and FcaR in neutrophils) [44]. Scientific evidence to date is also relating the abundance of *L. reuteri* and *L. rhamnosus* GG to some immunomodulatory effects that can be beneficial for allergenic populations affected by atopic dermatitis, food allergies, and asthma [45,46,47,48].

Although fucoidan has been shown to be effective as a prebiotic, emphasizing the positive growth of anti-allergy microbiota, the origin of fucoidan (*F. vesiculosus*, *F. serratus*, *F. spiralis*, *Fucus evanescens*, *Saccharina latissima*, *Ulva lactuca*, *M. pyrifera*, *U. pinnatifida*, and *Cladophora* sp.) and its chemical structure seriously determines its functional effect.

In 2017, fucoidan achieved the generally recognized as safe status (GRAS) provided by the U.S. Food and Drug Administration (FDA), which meant it could be used as a food ingredient in snacks, dairy products, soups, and baked goods, among others [49]. The maximum recommended level of consumption was fixed at 240 µg/mL per serve. However, no toxicological negative effects were found in animal studies using daily dosages of fucoidan in the range of 100–2000 µg/mL. Therefore, extended research is required to find the best fucoidan option to ensure the desired effects from novel formulated commodities, accomplishing the criteria of using the minimum dosage with the maximum desired effect (in this case prebiotic).

According to the obtained data in point 3.1 (in vitro studies of the fucoidan prebiotic effect in reference media) and the FDA fucoidan GRAS recognition and toxicological studies [49], 500 µg/mL of fucoidan has been selected as a conservative concentration in the search for an equilibrium between effectiveness and health safety.

### 3.2. The Impact of Fucoidan Addition to L. retueri and L. rhamnosus Growth/Viability in a New Formulated Synbiotic Yogurt

**Figure 3:**
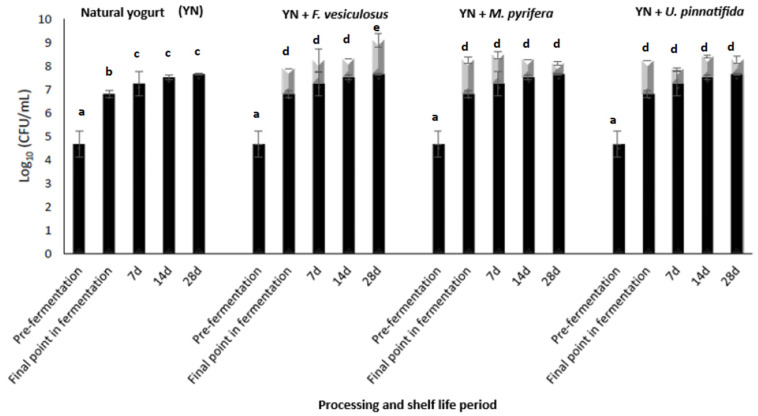
Progression of bacterial counts in yogurt (*L. delbrueckii* and *S. thermophilus*) during the processing (pre-fermentation t = 0 h; final point in fermentation, t = 4 h), and shelf life of samples (t = 7, 14, and 28 days), formulated and supplemented with and without fucoidan from different origins (added at 500 μg/mL): *Fucus vesiculosus*, *Macrocystis pyrifera*, and *Undaria pinnatifida*. (a–e) Superscript letters indicate significant differences between bars values. Grey bars represent prebiotic effect due to fucoidan addition.

Firstly, natural yogurt supplementation with fucoidan was studied in terms of total count viability (including *L. delbrueckii subs bulgaricus* and *S. thermophilus*) (Figure 3). In natural yogurt (YN) (control), an increase equivalent to 2 log_10_ cycles is observed in total counts (*L. delbrueckii subs bulgaricus* + *S. thermophilus*) after 4 h of fermentation (final point of fermentation), increasing the mean bacterial load from 4.67 log_10_ (t = 0 h) to 6.80 log_10_ (post-fermentation) at 42 °C (pH < 4.6). The fucoidan addition to the media (500 µg/mL) before starting fermentation increases lactic acid bacteria (BAL) in yogurt, with a stimulating growth effect equivalent to 1.5 log_10_ cycles in yogurt supplemented with Fucus vesiculosus and *Undaria pinnatifida*, and with a maximum increment of 1 log10 cycles in yogurt formulated with *Macrocysits pyrifera*.

The bacterial load in the final product is generally maintained close to 8 log10 cycles during the shelf life of yogurts, being for all fucoidan supplemented yogurts an observed increment in bacterial load close to 1–1.5 log_10_ cycles. The maximum prebiotic effect of fucoidan addition in yogurt was detected for Fucus vesiculosus-formulated yogurts that remain close to 9.10 ± 0.27 log_10_ cycles after 28 d, 4 °C (meanwhile YN is close to 7.64 ± 0.04 log_10_ cycles at the end of the same period).

**Figure 4:**
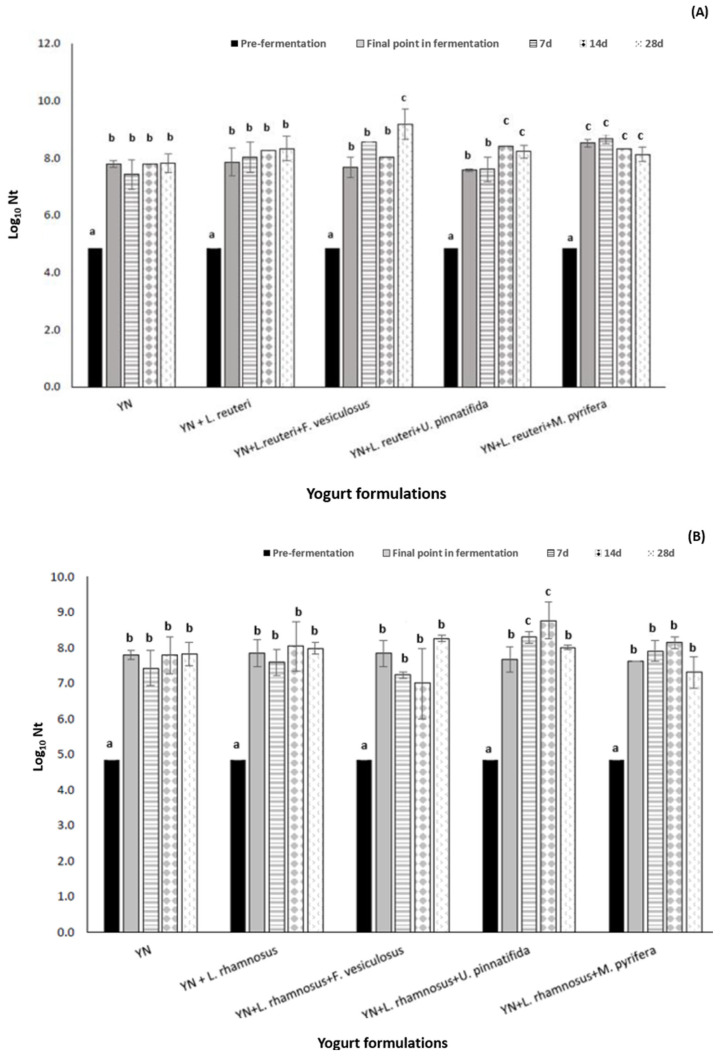
Study of viability/growth behavior of *L. reuteri* (**A**) and *L. rhamnosus* GG (**B**) in novel formulated yogurts including fucoidan as a prebiotic agent (500 μg/mL), during a refrigerated storage period of 28 days (at 4 °C). (**A**) Natural yogurt, [YN]; natural yogurt formulated with *L. reuteri*, [YN + *L. reuteri*]; natural yogurt formulated with *L. reuteri* and supplemented with *F. vesiculosus*, [YN + *L. reuteri* + *F. vesiculosus*]; natural yogurt formulated with *L. reuteri* and supplemented with *Undaria pinnatifida*, [YN + *L. reuteri + U. pinnatifida*]; natural yogurt formulated with *L. reuteri* and supplemented with *Macrocystis pyrifera*, [YN + *L. reuteri + M. pyrifera*]. (**B**) Natural yogurt, [YN]; natural yogurt formulated with *L. rhamnosus* GG, [YN + *L. rhamnosus*]; natural yogurt formulated with *L. rhamnosus* and supplemented with *F. vesiculosus*, [YN + *L. rhamnosus* + *F. vesiculosus*]; natural yogurt formulated with *L. rhamnosus* and supplemented with *Undaria pinnatifida*, [YN + *L. rhamnosus + U. pinnatifida*]; natural yogurt formulated with *L. rhamnosus* and supplemented with *Macrocystis pyrifera*, [YN + *L. rhamnosus + M. pyrifera*]. (a–c) Superscript letters correspond to points with significant (*p*-value < 0.05) increase in bacterial growth between batches.

Regarding yogurt formulated including *L. reuteri* and *L. rhamnosus* GG, the viability of both probiotics in yogurt is maintained stable after 28 days in the presence of the own microbiota in yogurt, L. *delbrueckii* subsp. *bulgaricus* and *S. thermophilus* (Figure 4). The prebiotic effect of fucoidan on probiotic bacteria growth in novel formulated yogurts was tested during the complete process of yogurt elaboration and post-fermentation refrigerated storage (up to 28 days). The results reveal a maximum of 1 log_10_ cycle increase in *L. reuteri* and/or *L. rhamnosus* counts in yogurt depending on the fucoidan origin (*p*-value < 0.05) added, and the storage time. Fucoidan from *M. pyrifera*, that showed the best prebiotic potential on *L. reuteri* and *L. rhamnosus* growth in previous in vitro studies (see Section 3.1.), showed in yogurt a significant and specific stimulating growth potential for *L. reuteri* (close to a 1 log_10_ cycle increase compared to *L. reuteri* growth in not-supplemented yogurt). In spite of this, during the progression of shelf life in yogurt, these values are progressively equated to *L. reuteri*-detected values in the other yogurt batches. For *L. reuteri*, fucoidan from F. vesiculosus also showed a significant prebiotic effect, guaranteeing *L. reuteri* final counts close to 9.17 ± 0.3 log_10_ cycles after 28 days of refrigeration compared to the viability of *L. reuteri* detected in YN after 28 days of refrigerated storage, 8.30 ± 0.15 log_10_ cycles.

In the case of *L. rhamnosus* GG growth and viability on yogurts, *U. pinnatifida* showed the best result as a prebiotic in yogurt. Just after 14 days of incubation, the final *L. rhamnosus* GG load was close to 8.78 log_10_ cycles, compared to counts observed in YN (7.93 log_10_ cycles).

According to the present results, it can be concluded that the supplementation of yogurt with fucoidan enhances the viability of the yogurt’s own bacteria (by 1.5–2 log_10_ cycles) and maintains the viability of probiotics *Limosilactobacillus reuteri* and *Lacticaseibacillus rhamnosus* GG, even increasing their growth close to 1 log_10_ cycle, with multiple positive associated health effects. Prabhurajeshwar et al. [50] demonstrated that different *Lactobacillus* spp. isolated from different yogurt samples have excellent resistance at low pH and at high concentrations of NaCl and bile acids, characteristics of the human gastrointestinal tract, which made these *Lactobacillus* spp. excellent probiotics. In addition, they showed that these bacteria present in the yogurt had excellent antibiotic properties against some pathogens of the human intestine. Although probiotic fermented milks—among them yogurts—are the most popular and investigated probiotic functional foods, specifically for yogurt, critical bacterial count values should be maintained until the end of the shelf life of the product to ensure its functionality and bioactivity.

To our knowledge, only a few studies have been published regarding the research on yogurt development involving *L. rhamnosus* and *L. reuteri* [51,52,53], but no one has been reported formulating a prebiotic/probiotic yogurt that includes fucoidan as a stimulating bacterial growth agent. In yogurt, the higher sensitivity of *L. reuteri* to survive is recognized, *L. rhamnosus* being more stable as regards the shelf life of the product. [53]. In comparison with other prebiotic agents like inulin, concentrations of this prebiotic in the range 6 to 20 mg/mL have been added to fermented milks in order to enhance *L. reuteri* or *L. rhmanosus* growth [54,55].

According to the obtained results in the present study, *L. reuteri* and *L. rhamnosus* GG maintain final values close to 10^7^ CFU/g in yogurt, during the complete shelf life of yogurts, according to the recommendations of the International Dairy Federation (must contain at least 10^6^ viable probiotic bacteria per gram or milliliter of product at the time of consumption). Moreover, it can be confirmed from the present results that the viability of both probiotics in yogurt generally benefits from the addition of fucoidan.

The synbiotic products market holds promising prospects for the coming years. A synbiotic is defined as “a combination of probiotics and prebiotics that beneficially affects the host by improving the survival and implantation of selected live microbial strains in the gastrointestinal tract” [53]. The market size is projected to reach USD 1116.9 million by 2033, driven by scientific advances and innovation focused on the development of novel synbiotic food products.

In this context, the novel yogurt formulation proposed in the present study—based on synbiotic combinations of *Limosilactobacillus reuteri* + fucoidan or *Lacticaseibacillus rhamnosus* GG + fucoidan—offers a dual benefit: (i) on one hand, *L. reuteri* and *L. rhamnosus* have been associated with the alleviation of atopic dermatitis, food allergy-related inflammatory responses, Crohn’s disease, ulcerative colitis, rheumatoid arthritis, and acute gastroenteritis caused by rotaviruses; (ii) on the other hand, fucoidan is recognized for its anticoagulant and antithrombotic properties, as well as its antimicrobial, antitumor, and immunomodulatory activities, in addition to antioxidant, lipid-lowering, anti-inflammatory, gastroprotective, and hepatoprotective effects. According to the obtained results, the present developed product could be an option as a future contribution to mitigate allergenic symptomatology [56].

## 4. Conclusions

Fucoidan derived from *Macrocystis pyrifera* and *Undaria pinnatifida* demonstrated the most effective results in promoting the growth of probiotics *L. rhamnosus* GG and *L. reuteri* in yogurt formulations. In fucoidan-supplemented yogurts (500 µg/mL), the levels of *L. reuteri* and *L. rhamnosus* remained near 10^7^ CFU/g. The native yogurt microbiota (*Streptococcus thermophilus* and *Lactobacillus delbrueckii*) remained at levels close to 10^8^ CFU/g at the time of consumption.

In this context, the present study is the first to demonstrate the potential of fucoidan to enhance the viability of key probiotic bacterial strains—*L. reuteri* and *L. rhamnosus* GG—in a real food matrix such as yogurt, and provides a comprehensive view of the potential of this nutraceutical ingredient for the development of tailored functional foods. These products could be targeted toward specific populations, such as individuals requiring preventive or complementary treatment for allergic conditions.

Further in vivo studies are necessary to determine appropriate dosages and to confirm the safety of regular fucoidan consumption, whether through diet or as a pharmacological product.

## Figures and Tables

**Table 1:** Kinetic parameter values obtained from Gompertz fit of *Limosilactobacillus reuteri* and *Lacticaseibacillus rhamnosus* GG growth curves.

Microbial Strain	Fucoidan Concentration (µg/mL)	A	C	B	M	R^2^-Adjusted	RMSE	ʎ	µ_max_	*Af*
*Fucus vesiculosus*										
*L. reuteri*	0 (Control)	6.31 ± 0.19	2.72 ± 0.27	0.15 ± 0.04	9.04 ± 1.31	0.98	0.08	2.23 ± 1.23 ^a^	0.15 ± 0.03 ^a^	1.03
	100	6.97 ± 0.87	7.28 ± 1.25	0.18 ± 0.01	7.23 ± 0.43	0.96	0.19	0.43 ± 0.67 ^b^	0.48 ± 0.02 ^b^	1.19
	2000	4.20 ± 0.12	15.98 ± 0.67	0.21 ± 0.01	4.12 ± 1.22	0.93	0.18	0.85 ± 0.11 ^b^	1.23 ± 0.04 ^c^	1.18
										
*L. rhamnosus* GG	0 (Control)	6.37 ± 0.09	2.76 ± 0.08	0.13 ± 0.01	6.91 ± 1.15	0.98	0.09	0.68 ± 0.23 ^a^	0.13 ± 0.07 ^a^	1.03
	100	5.03 ± 0.45	12.72 ± 0.24	0.14 ± 0.04	5.11 ± 1.43	0.91	0.23	0.99 ± 0.14 ^a^	1.55 ± 0.11 ^b^	1.09
	2000	3.11 ± 0.41	13.68 ± 0.24	0.25 ± 0.04	6.23 ± 2.22	0.92	0.19	−0.24 ± 0.06 ^b^	1.34 ± 0.22 ^c^	1.11
*Undaria pinnatifida*										
*L. reuteri*	0 (Control)	0.03 ± 0.01	8.01 ± 0.32	0.13 ± 0.01	0.5 ± 0.05	0.99	0.11	−24.1 ± 1.12 ^a^	0.37 ± 0.03 ^a^	1.22
	100	2.17 ± 0.22	5.76 ± 0.87	0.24 ± 0.02	4.71 ± 0.36	0.99	0.07	−7.12 ± 1.23 ^a^	0.50 ± 0.01 ^b^	1.12
	2000	0.59 ± 0.06	7.18 ± 0.25	0.09 ± 0.04	1.71 ± 0.82	0.97	0.21	−38.1 ± 2.58 ^a^	0.53 ± 0.01 ^b^	1.03
										
*L. rhamnosus* GG	0 (Control)	6.35 ± 0.56	8.22 ± 0.36	0.13 ± 0.01	6.81 ± 0.11	0.98	0.09	−0.50 ± 0.05 ^a^	0.20 ± 0.03 a	1.04
	100	4.91 ± 0.08	8.56 ± 0.25	1.04 ± 0.37	3.15 ± 0.17	0.98	0.10	−1.75 ± 0.36 ^b^	0.65 ± 0.01 ^b^	1.15
	2000	4.90 ± 0.32	9.23 ± 0.11	0.51 ± 0.08	7.75 ± 0.22	0.99	0.09	−3.16 ± 0.85 ^b^	1.26 ± 0.01 ^b^	1.23
*Macrocystis pyrifera*										
*L. reuteri*	0 (Control)	3.99 ± 0.71	6.81 ± 0.51	0.25 ± 0.03	23.7 ± 1.23	0.98	0.16	13.10 ± 1.74 ^a^	0.75 ± 0.03 ^a^	1.08
	100	3.22 ± 0.12	8.36 ± 1.32	0.66 ± 0.12	3.63 ± 0.87	0.98	0.09	−0.51 ± 1.21 ^b^	2.55 ± 0.01 ^b^	1.03
	2000	3.53 ± 0.08	8.61 ± 0.15	3.37 ± 1.22	3.33 ± 0.62	0.98	0.15	2.78 ± 1.23 ^b^	5.71 ± 0.07 ^c^	1.11
										
*L. rhamnosus GG*	0 (Control)	4.75 ± 0.66	6.31 ± 0.12	0.35 ± 0.03	5.67 ± 0.73	0.97	0.011	−2.39 ± 0.26 ^a^	0.29 ± 0.03 ^a^	1.25
	100	5.22 ± 0.24	8.69 ± 1.21	0.36 ± 0.05	9.11 ± 1.21	0.99	0.06	2.80 ± 0.15 ^b^	0.43 ± 0.01 ^b^	1.04
	2000	5.61 ± 0.13	8.23 ± 0.65	0.23 ± 0.12	9.67 ± 1.32	0.99	0.06	3.36 ± 0.33 ^b^	0.52 ± 0.01 ^b^	1.01

A, the lower asymptote in the curve (log_10_ (CFU/mL); C, difference between the curve asymptotes (log_10_ CFU/mL); B, relative growth rate when t = M ((log_10_ CFU/mL)/h); M, the elapsed time until the maximum growth rate is reached (h); R^2^-adjusted and RMSE (root mean square error) used as a measure of the goodness of fits; *λ* (h). is the lag phase duration; *μ_max_* is the maximum specific growth rate ((log_10_ (cfu/mL))/h), *Af*, accuracy factor [31]. Superscript letters (a–c) refer to significant differences (*p*-value < 0.05) between values in rows (treatments with fucoidan 0–2000 µg/mL).

## Data Availability

The original contributions presented in this study are included in the article. Further inquiries can be directed to the corresponding authors.
